# An Icy Vista from a Golden Age

**DOI:** 10.3201/eid2412.AC2412

**Published:** 2018-12

**Authors:** Byron Breedlove

**Affiliations:** Centers for Disease Control and Prevention, Atlanta, Georgia, USA

**Keywords:** art science connection, emerging infectious diseases, An Icy Vista from a Golden Age, Winter Landscape with Ice Skaters, Hendrick Avercamp, epidemics, weather, climate, Little Ice Age, plague, public health, zoonoses, about the cover

**Figure:**
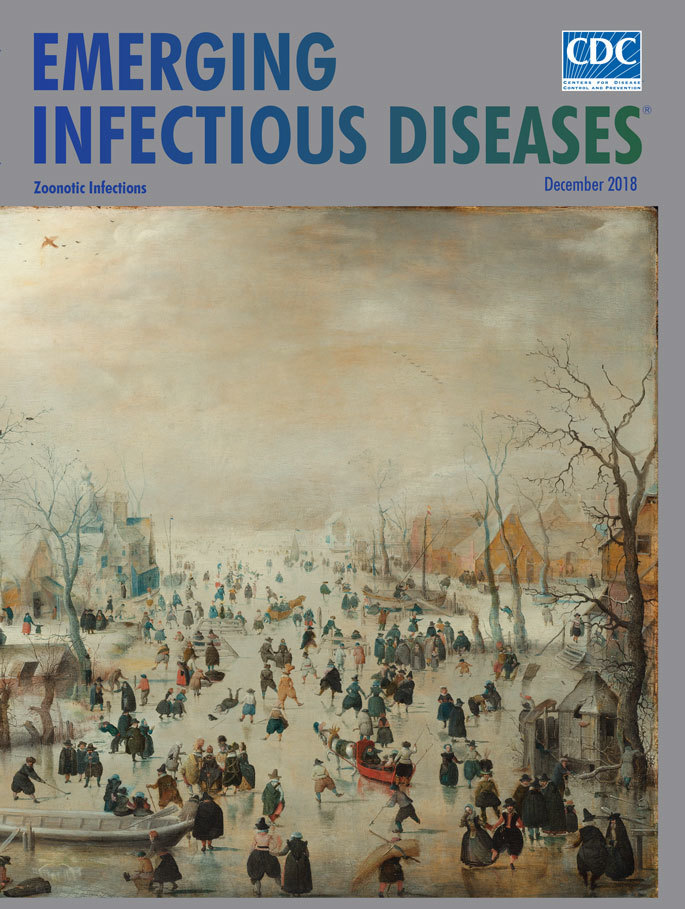
**Hendrick Avercamp (1585–1634), *Winter Landscape with Ice Skaters*** (c. 1608). Oil on panel, 30.4 in x 52 in/77.3 cm x 131.9 cm. Digital image courtesy of Rijksmuseum, Amsterdam.

“No other nation in the world looks forward to an icy season the way the Dutch do,” wrote Wim Pijbes and Earl A. Powell III in the preface to *Hendrick Avercamp: Master of the Ice Scene.* Wintery weather predominated across most of Europe during the Little Ice Age, which started around 1300 and lasted for about 350 years. The atypically cold, harsh weather affected agriculture, health, commerce, emigration, and social stability, precipitating unrest, revolt, warfare, and migration. Throughout this time, epidemics of plague flared across Europe, and malaria persisted despite frigid conditions. 

The coldest period of the Little Ice Age roughly overlapped the 90 years of the Dutch Golden Age. Unlike most of Europe, the Dutch Republic, which preceded the Netherlands of today, thrived during the icy 17th century. Despite its small size and limited natural resources, the country achieved stability and prosperity, chiefly because of its unmatched commercial fishing and trading fleets and its agricultural practices. That confluence of “political, economic, religious, and social circumstances created a unique and fruitful climate for the arts,” according to the National Gallery of Art, which also notes, “A remarkable number of pictures of extraordinary quality were produced during the Dutch Golden Age.” 

Perhaps less known than some of his contemporaries, Hendrick Avercamp is nonetheless considered to be among the great masters of that time. Avercamp painted vibrant, detailed winter landscapes during the first third of the 17th century. The United Sates National Gallery of Art states that he “did not invent the winter landscape, but he was the first artist to specialize in this quintessential Dutch subject.” 

Details of Avercamp’s life are sparse. He was born in Amsterdam in 1585, though the next year his family relocated to the smaller city of Kampen. His father, Barent, served as the town apothecary and later as its doctor until he died of the plague in 1602. His first art teacher and one of his brothers also died of the plague during that frigid 17th century. 

Apparently, Avercamp could not speak and was known as “the Mute of Kampen.” He came from a learned family: his father and brothers were physicians and apothecaries, and his grandfather and uncle were headmasters of a prestigious Latin school. Avercamp probably lived in the house that his mother Beatrix owned. Art historian Arthur K. Wheelock Jr. observed that “There, in relative isolation from the mainstreams of Dutch art, he devoted himself almost entirely to the painting of winter scenes and specifically to depictions of crowds of people engaging in a wide range of activities on frozen rivers.” In total, Avercamp produced about a hundred paintings of winter scenes.

Avercamp completed *Winter Landscape with Ice Skaters,* this month’s cover art, following the exceptionally frigid winter of 1607–1608. The painting, which is his largest known work, reveals an icy fog cloaking the winter sun and the edges of the village. A stark filigree of bare tree limbs claws toward the winter sky. The artist achieved an impression of depth by fading colors and blurring­ shapes in the distance. His placement of buildings and trees that flank the frozen canal also guides viewers toward the misty horizon. Pieter Roelofs, a curator at Rijksmuseum, Amsterdam, observes that Avercamp’s use of vantage points and perspective served to “create a more natural space so that the viewer is involved more directly in the events on and around the ice.” 

The Rijksmuseum offers additional details: “The high vantage point of this painting turns it into a sampler of human―and animal―activity during a harsh winter. Hundreds of people are out on the ice, most of them for pleasure, others working out of dire necessity.” A crowd of busy figures is scattered across the canvas. Among the working class, a beggar seeks bread, an angler balances a long trident and grips the day’s catch, and a brewery worker fetches water from a hole cut into the ice. Knots of people conversing, couples skating, and sportsmen playing kolf on the ice (a hybrid golf and hockey) reveal that for many others this is a day for recreation and socializing. 

Animals scattered throughout the painting include several horses pulling sledges; birds hovering in the silvery air or clinging to bare branches or sloping rooftops; domesticated fowl pecking for food; and a dog scampering over the ice. Roelofs notes, “Avercamp did not concentrate solely on winter fun and games and the sunny side of the cold season.” For example, he placed crows and a dog feasting on a horse carcass in the foreground of this painting. 

Environmental history professor Dagomar Degroot comments on the Little Ice Age: “Changing weather patterns altered the range of insects that carried pathogens, bringing new and deadly ailments to the previously unexposed. Because malnourished bodies have weak immune systems, farmers and their livestock soon fell sick. Refugees from the famine-stricken countryside spread disease to cities, where epidemic outbreaks often inflicted a fearsome toll.” *Winter Landscape with Ice Skaters* provides a glimpse of village life during Europe’s Little Ice Age and may stimulate viewers to consider the interrelationship of climate and human, animal, and environmental health. 
